# Complete Genome Sequencing of Eight Brucella abortus Biovar 1 Strains Isolated from Water Buffalo

**DOI:** 10.1128/genomeA.00179-18

**Published:** 2018-03-29

**Authors:** Rubina Paradiso, Massimiliano Orsini, Marita Georgia Riccardi, Bianca Cecere, Anna Cerrone, Cesare Cammà, Maria Luisa Chiusano, Giorgio Galiero, Giorgia Borriello

**Affiliations:** a Istituto Zooprofilattico Sperimentale del Mezzogiorno, Portici, Italy; b Istituto Zooprofilattico Sperimentale G. Caporale, Teramo, Italy; c Dipartimento di Agraria, Università degli Studi di Napoli “Federico II,” Portici, Italy

## Abstract

Brucellosis is a zoonotic disease caused by bacteria of the genus Brucella. The disease is endemic in many areas, causing chronic infections responsible for reproductive disorders in infected animals. Here, we present eight complete genome assemblies of eight Brucella abortus strains isolated from water buffaloes farmed in the Campania region.

## GENOME ANNOUNCEMENT

Brucellosis is a zoonotic infection caused by bacteria of the genus Brucella (1). This pathogen is transmitted from animals to humans by ingestion of contaminated food, direct contact with infected animals, or inhalation of aerosols. Brucella organisms, which are small aerobic intracellular coccobacilli, localize in the reproductive organs of host animals, causing abortions and sterility.

Brucella abortus is widespread throughout the world and particularly in southern Italy (2), where despite various strategies for its control and eradication, every year there are new outbreaks of the disease.

Brucella virulence is highly complex and characterized by extremely efficient adaptation mechanisms providing the bacteria with the ability to evade host immune recognition and to manipulate key aspects of host cell physiology (1).

Many studies are still necessary to define the epidemiology of brucellosis in water buffalo and understand the molecular mechanisms associated with its virulence. Therefore, in this study we selected eight B. abortus biovar 1 strains, isolated from water buffaloes farmed in the Caserta and Salerno districts, for sequencing and genetic characterization.

Strains were inoculated onto Brucella medium base (Oxoid) and incubated at 37°C in 5 to 10% CO_2_, and DNA was purified with the QIAamp DNA minikit (Qiagen), as described by the manufacturer. For sequencing we used approximately 50 to 100 ng of genomic DNA, to produce fragments of 400 bp in length. Libraries were prepared using an Ion Shear Plus reagent kit (Life Technologies) for the Ion Torrent sequencing platform. The generated sequencing reads were checked using FastQC, and low-quality sequences were removed by using PRINSEQ lite. High-quality sequences were successfully assembled using SPAdes (3) version 3.8.2, improved with Pilon 1.8, and manually checked to close eventual gaps. The draft assemblies of the eight strains were annotated using the NCBI Prokaryotic Genome Annotation Pipeline version 4.2.

The Brucella genome has two circular chromosomes. For each strain an average of 96.3% ± 3.7% of the obtained sequences was assembled with a mean contig number of 41 ± 3.4% among assemblies. For each strain, we identified all coding genes and those coding for proteins involved in virulence mechanisms. By use of Bowtie2 version 2.2.9, reads were aligned to the genome of the reference strain B. abortus A13334 (GenBank accession numbers NC_016795 and NC_016777). The number of single nucleotide polymorphisms (SNPs) was determined using MUMmer 3.0. This study highlights the complexity of the Brucella genome and represents a basis for further studies aiming toward a better comprehension of the virulence mechanisms of this pathogen.

### Accession number(s).

The whole-genome sequences of B. abortus biovar 1 strains were deposited in GenBank under BioProject number PRJNA400357, with the accession numbers listed in Table 1.

**TABLE 1:** Main characteristics and GenBank accession numbers of the eight *B. abortus* biovar 1 genomes assembled

Strain	No. of genes	No. of SNPs	No. of virulence genes	GenBank accession no.	
				Chromosome 1	Chromosome 2
9510	3,297	1,114	27	CP023308	CP023309
84573	3,396	1,258	27	CP023241	CP023242
7863	3,397	1,330	27	CP023229	CP023230
49839	3,363	1,174	30	CP023219	CP023220
40046	3,392	1,327	27	CP023215	CP023216
33295	3,399	1,758	27	CP023213	CP023214
28375	3,316	1,290	28	CP023211	CP023212
14330	3,335	1,227	27	CP023243	CP023244
